# Minimally Invasive Aortic Valve Replacement in a Patient With Interrupted Hepatic Inferior Vena Cava and Duplicated Inferior Vena Cava: A Case Report

**DOI:** 10.7759/cureus.87009

**Published:** 2025-06-29

**Authors:** Gentaku Hama, McAndrew Merlini, Keijiro Mitube, Tomoya Ohshiro

**Affiliations:** 1 Cardiovascular Surgery, Sapporo Heart Center, Sapporo, JPN

**Keywords:** aortic valve replacement, duplicated ivc, inferior vena cava anomaly, interrupted hepatic ivc, minimally invasive cardiac surgery

## Abstract

We report a case of a 72-year-old female patient with severe aortic regurgitation and a rare congenital anomaly involving duplicated inferior vena cava (IVC) and interruption at the hepatic segment. She underwent minimally invasive cardiac surgery-aortic valve replacement (MICS-AVR) via right anterior thoracotomy. Venous cannulation was performed through the right internal jugular vein and right femoral vein, while arterial access was achieved through the right femoral artery. Despite suboptimal venous return, cardiopulmonary bypass (CPB) was initiated, and the procedure was completed successfully with hypothermic support. The patient recovered uneventfully and was discharged on postoperative day six. This case highlights the importance of thorough preoperative imaging and intraoperative vigilance when dealing with vascular anomalies in MICS.

## Introduction

Inferior vena cava (IVC) anomalies, including duplicated IVC and interrupted hepatic IVC, are rare congenital conditions with a prevalence of approximately 0.6% in the general population [1]. These anomalies are often asymptomatic and incidentally found during imaging studies. However, they can pose significant challenges during minimally invasive cardiac surgery (MICS) procedures. The IVC develops between the 4th and 8th weeks of gestation through a complex process involving the postcardinal, subcardinal, and supracardinal veins. Disruptions in this process may result in variations such as duplication or interruption [2,3]. In this report, we present a case of successful MICS-aortic valve replacement (AVR) in a patient with duplicated IVC and interruption at the hepatic segment.

## Case presentation

**Figure 1:**
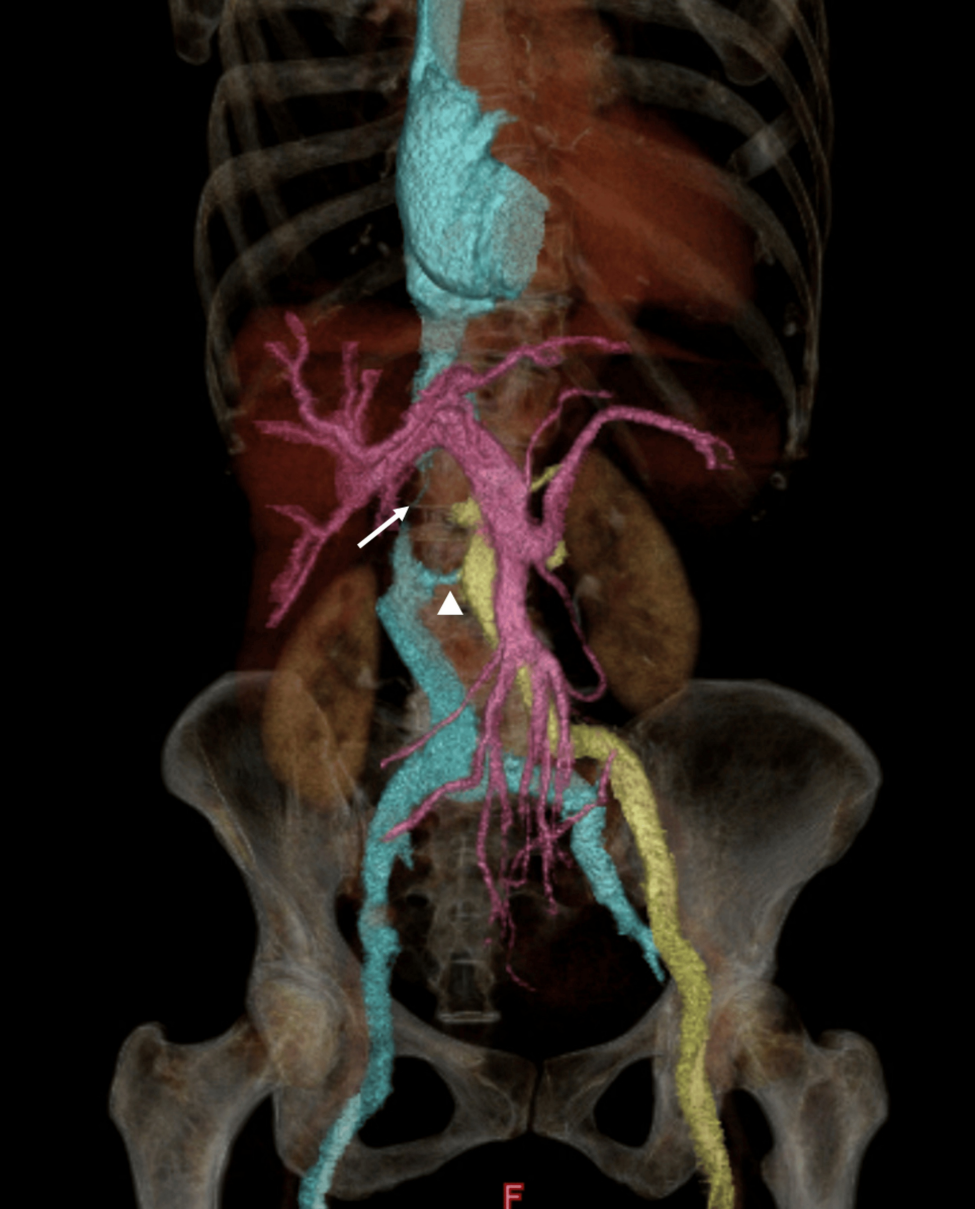
Three-dimensional CT reconstruction of venous system anomalies IVC: inferior vena cava 3D CT reconstruction of duplicated IVC and hepatic segment interruption. Yellow indicates the left-sided IVC, blue indicates the right-sided IVC, and red indicates the hepatic vein. The arrow highlights the interruption of the hepatic segment, the triangle indicates the left renal vein, through which the left-sided IVC drains into the right-sided system

**Figure 2:**
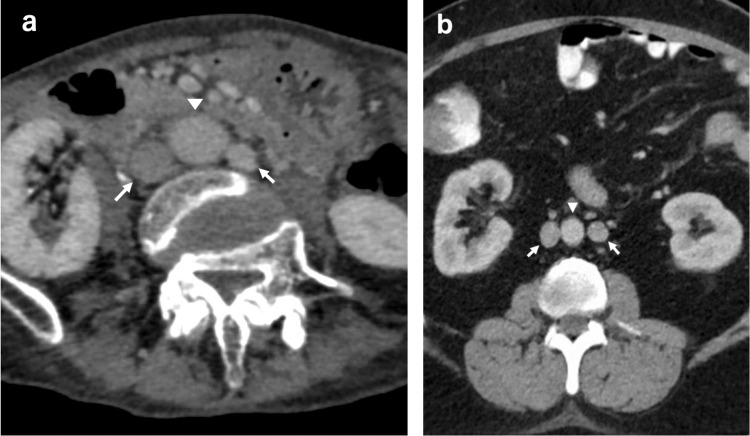
Axial CT images IVC: inferior vena cava (a) Our case shows duplicated inferior vena cava (IVC), with arrows indicating the right- and left-sided IVC, and the triangle indicating the abdominal aorta. (b) Reference image reproduced from Li et al. [1], showing a similar pattern of duplicated IVC

A 72-year-old female patient presented with exertional dyspnea and an abnormal electrocardiogram suggestive of left ventricular hypertrophy during a routine health examination. She had no prior cardiovascular history or known congenital anomalies. Symptoms had persisted for several months. Transthoracic echocardiography revealed severe aortic regurgitation (regurgitant volume: 66 mL) with left ventricular dysfunction (LVDd/Ds, 61/47 mm; EF, 46%). Contrast-enhanced CT revealed a duplicated IVC with an interrupted hepatic segment. Figure 1 shows 3D reconstructed imaging. Axial CT images (Figure 2a) demonstrate duplicated IVC, while Figure 2b, adapted from Li et al. [1], shows a reference image of duplicated IVC.

**Figure 3:**
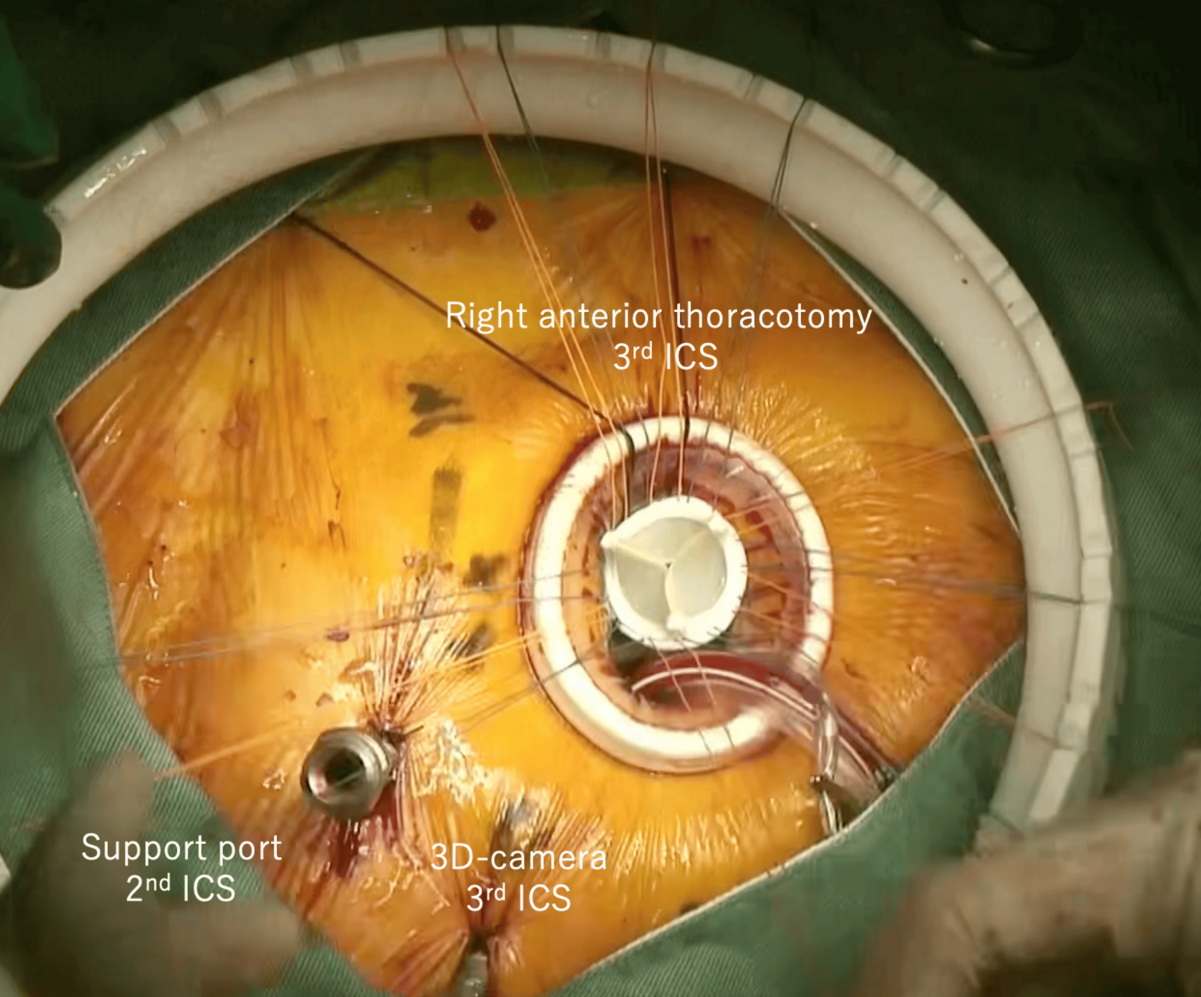
Port placement for MICS-AVR in this case MICS-AVR:  minimally invasive cardiac surgery-aortic valve replacement; ICS: intercostal space Intraoperative view showing the typical port configuration used during MICS-AVR: right anterior thoracotomy in the 3rd ICS, support port in the 2nd ICS, and 3D camera port in the 3rd ICS. This setup was not modified despite the vascular anomaly, as venous access was achieved with an adjusted cannulation strategy rather than changes in port placement

MICS-AVR was performed through a right anterior thoracotomy using a 3D thoracoscopic system. Figure 3 illustrates the surgical setup. Venous cannulation was achieved via the right internal jugular vein (19 Fr) and right femoral vein (17 Fr), with shallow placement of the femoral cannula due to the IVC interruption. Arterial cannulation was via the right femoral artery. Because drainage was insufficient (maximum flow, 2.6 L/min; ideal, 3.3 L/min for BSA 1.37 m²), mild hypothermia (30°C) and vacuum-assisted venous drainage were used to support perfusion. A 23 mm Inspiris Resilia valve (Edwards Lifesciences, Irvine, CA, USA) was implanted. Venous cannulae (Bio-Medicus NextGen, Medtronic, Minneapolis, MN, USA) were used. Left ventricular venting was performed via the left upper pulmonary vein. The postoperative course was uneventful. She was discharged on postoperative day 6. At three-month follow-up, valve function remained stable with no symptom recurrence.

## Discussion

Li et al. provided a comprehensive review of IVC variants using cross-sectional imaging, which is essential in preoperative planning for MICS [1]. IVC anomalies, although frequently asymptomatic, can present serious technical challenges during venous cannulation. In our case, identification of duplicated IVC with hepatic interruption necessitated a personalized cannulation strategy. Unlike standard AVR procedures, where single femoral venous cannulation is sufficient, we employed a dual-drainage approach. A second cannula was added via the right internal jugular vein, and the femoral cannula was deliberately positioned shallowly below the hepatic interruption.

Previous literature has reported cases of failed cannulation or retroperitoneal hematomas due to unrecognized IVC anomalies [4,5]. In a particularly severe case, fatal hemorrhage occurred during femoral cannulation for venovenous ECMO in a patient with duplicated IVC [6]. Had conventional cannulation been attempted in our case, serious complications might have ensued.

Transesophageal echocardiography (TEE) is critical for verifying cannula positioning. However, anomalous venous pathways can cause guidewires or cannulas to deviate from expected routes, making them difficult to trace. Although fluoroscopy was not required intraoperatively in this case, it remains a valuable modality when visualization is challenging [7].

MICS demands precision in vascular access. While offering advantages in aesthetics and recovery, the presence of IVC anomalies increases procedural complexity and risk [8]. In our case, despite reduced venous return, mild hypothermia allowed safe completion of cardiopulmonary bypass (CPB). Alternative drainage options, such as direct right atrial cannulation or additional cannulation via the left femoral vein, should also be considered.

## Conclusions

This case illustrates the importance of recognizing IVC anomalies in MICS. Contrast-enhanced CT enabled detection of duplicated and interrupted IVC, which informed a modified cannulation plan, ensuring procedural safety. Surgeons should remain vigilant for such anomalies and consider preoperative imaging and alternative cannulation routes to minimize risks.
